# Promising minimally invasive treatment modalities for symptomatic temporomandibular joint disc displacement with reduction: a randomized controlled clinical trial

**DOI:** 10.1186/s12903-022-02579-3

**Published:** 2022-12-01

**Authors:** Nermeen A. Rady, Mariam M. Bahgat, Ahmed M. Abdel-Hamid

**Affiliations:** Prosthodontics Department, Faculty of Dentistry, Alexandria University, Azarita, 21526 Alexandria Egypt

**Keywords:** Temporomandibular disorders, Internal derangement, Botulinum toxin, Photobiomodulation, Lateral pterygoid muscle, DC/TMD, MRI, Visual analogue scale, Disc position, Joint space index

## Abstract

**Background:**

Pain and clicking are the primary complaints in patients suffering from temporomandibular joint disc displacement with reduction (DDwR), negatively affecting the patients' quality of life, making the treatment essential. This prospective randomized controlled trial (RCT) was conducted to evaluate the effectiveness of botulinum toxin type-A (BTX-A) and low level laser therapy (LLLT) in comparison to anterior repositioning appliance (ARA) for the treatment of DDwR.

**Methods:**

A total of 27 patients were randomly allocated to 3 groups; ARA (control group), BTX-A, and LLLT; with 9 patients each. All patients were evaluated before and 3 months after the treatment using a visual analogue scale (VAS) and magnetic resonance imaging (MRI).

**Results:**

At 3 months follow-up, all groups showed a significant reduction in pain assessed by VAS (*P* = 0.007). Measured on MRI, there was a significant improvement in disc position and joint space index (JSI) in BTX-A group (*P* < 0.001, *P* = 0.011) and LLLT group (*P* = 0.002, *P* = 0.017) in comparison to the control group (*P* = 0.087, *P* = 0.066) respectively. As for time of recovery, a statistically significant difference was observed in BTX-A group (*P* < 0.001) and LLLT (*P* < 0.001) group in comparison to ARA group, which showed the most prolonged duration for reduction of DDwR symptoms.

**Conclusion:**

We concluded that BTX-A and LLLT could be considered effective alternative treatment modalities to ARA regarding reducing joint pain, clicking, and improving disc position in patients with symptomatic DDwR.

**Trial registration:**

This prospective double-blinded RCT has been registered at ClinicalTrials.gov with identification number: NCT05194488, 18/1/2022.

## Background

Aside from odontogenic pain, the pathology of orofacial pain is most commonly caused by temporomandibular disorders (TMDs). TMDs embrace a number of clinical conditions which involve the masticatory musculature, temporomandibular joint (TMJ), and associated structures [1]. TMDs are associated with joint sounds, restricted mouth opening, impairing functional mandibular movements, and negatively affecting the patient’s quality of life. Researchers generally agree that TMDs include myofascial pain, internal derangement (ID), and arthritis.ID is the most frequent type of TMDs in which the smooth joint functions are impaired with an abnormal disc position [1–3].

There is a lack of scientifically validated evidence about the etiology of ID. However, several factors have been suggested as etiological factors including parafunctional habits, alteration in the synovial fluid composition, abnormal activity of the lateral pterygoid muscle (LPM), trauma, and psychological stress [4–9].

**Fig. 1:**
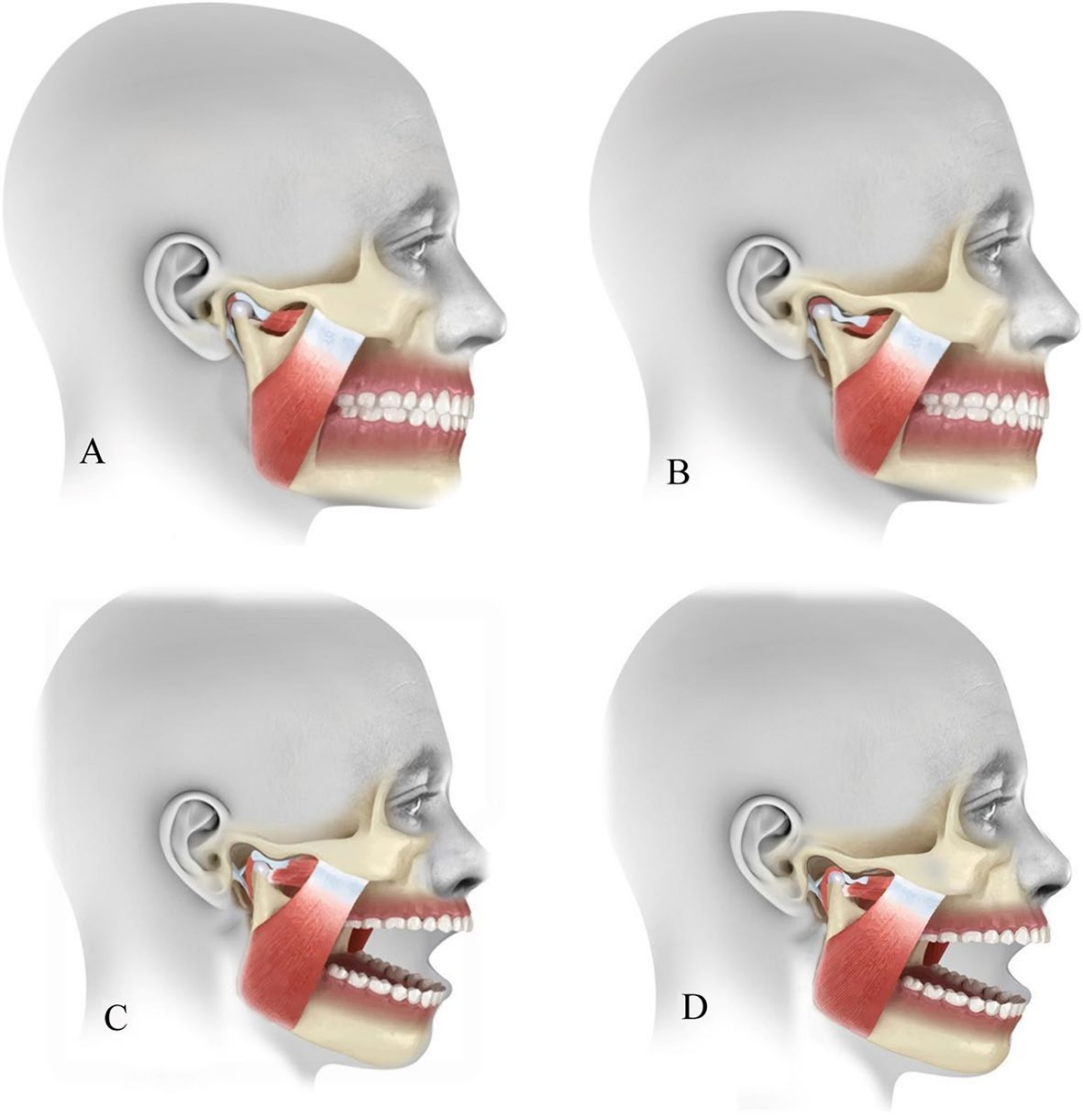
Illustrative diagram showing. **A.** normal TMJ, **B.** disc displaced in closed mouth, **C.** disc is reduced in open mouth position (DDwR), **D.** disc is not reduced in open mouth position

According to the Diagnostic Criteria for TMDs (DC/TMDs), IDs are classified into: DDwR, DDwR with intermittent locking, and disc displacement without reduction with or without limited mouth opening [2]. The most common type is DDwR with or without intermittent locking, characterized by clicking in the TMJ (Fig. 1A–D).

MRI provides information about articular disc position and morphology through high soft tissue resolution allowing assessment of TMJ anatomy and biomechanics through imaging in open and closed mouth positions with no ionizing radiation [10–12].

In DDwR, the disc is displaced anteriorly as a result of elongation of inferior retrodiscal lamina and discal collateral ligament with eccentric condylar position resulting in an abnormal disc-condyle relationship. Although the patient can maneuver the jaw position to regain the normal disc-condyle relationship, this movement results in joint pain, tenderness in associated masticatory muscles, and clicking [13].

The goal of treatment is to eliminate or reduce pain with restoring the normal TMJ function. Through the years, there has been a tremendous growing interest in minimally invasive approaches as first-line therapy. These approaches include cognitive behavioral therapy, pharmacotherapy, oral appliances, intra-articular injections, intramuscular injections as botulinum toxin, physical therapy including ultrasound, acupuncture, transcutaneous electrical nerve stimulation, and laser [14–19].

Among several oral appliances, several authors reported that ARA is advocated as a passive intervention to reduce the biomechanical load on the TMJ in symptomatic DDwR. This appliance guides the patient’s mandible forward until the disc is reduced, and this protruded position is indexed into the appliance to maintain a reduced disc and prevent impingement on the retrodiscal tissues [16–23].

Botulinum toxin (BTX) is an exotoxin produced from *Clostridium botulinum*. BTX blocks the release of acetylcholine at the presynaptic junction producing a temporary and dose-dependent decrease in muscle activity and the glands innervated without any systemic effects [24, 25]. In addition, BTX inhibits specific proteins that regulate the production of inflammatory mediators resulting in chronic pain relief. Moreover, BTX affects pain processing through reducing the central pain sensitization, which is the responsible mechanism for chronic pain [26]. Seven serotypes of BTX are given alphabetical letters from A to G with varying biochemical and pharmacological actions with different protein complex compositions. Although all BTX serotypes prevent acetylcholine release, they act on different intracellular proteins exhibiting differences in duration and effectiveness of action [27–29]. Regarding its therapeutic effects, BTX-A is the most effective and safest serotype with the most extended effect duration [26, 29, 30]. Several studies were conducted to test the effect of BTX-A as a novel therapy for treating myogenous TMDs, where it was injected in the temporalis and masseter muscles which was found effective for up to 6 months [31–34]. However, few studies investigated its effect on the management of DDwR when injected into the LPM [35, 36].

LLLT has been presented as an effective non-invasive physical treatment modality for TMDs. LLLT has lower energy output and its mechanism of action is based on light absorption, so it does not increase skin temperature. It typically uses a wavelength of range 630-1300 nm. The physiological effects of LLLT include photobiomodulation, anti-inflammatory and anti-edematous action, analgesic effect, and promoting tissue regeneration [37, 38].

Photobiomodulation occurs as the laser light reacts with beta growth factors and oxygenated molecules, so it stimulates the production of vascular endothelial growth factor, which improves blood circulation and vascularization [39]. Through its anti-inflammatory and anti-edematous action, LLLT increases adenosine triphosphate production, improves the local microcirculation, reduces the edema through encouraging early drainage of the interstitial fluid as a result of increasing lymphatic flow, and decreases the levels of prostaglandin E2 and cyclooxygenase-2 so consequently inflammation decreases [40].

As for the analgesic effect, LLLT increases the pressure-pain threshold through an electrolytic nerve fiber blocking action and causes a reduction in histamine and acetylcholine release with a decrease in bradykinin synthesis [41, 42].

LLLT promotes tissue regeneration by raising cell activity and adenosine triphosphate production. Moreover, it increases growth factors and cytokines release with acceleration in replication mechanisms which declines the oxidative phase and promotes cell repair [43]. Based on these mentioned therapeutic effects, systematic reviews suggested that LLLT is an effective physical therapy for managing TMDs [44, 45]. However, there is little evidence for its effectiveness in treating symptomatic DDwR.

To the authors’ knowledge, there is a lack of evidence in the literature comparing the effectiveness of BTX-A and LLLT in managing symptomatic DDwR. Therefore, this prospective RCT was conducted to evaluate the effectiveness of BTX-A and LLLT as emerging treatment modalities in comparison to ARA for the management of symptomatic DDwR. The null hypothesis was that there is no difference between BTX-A and LLLT compared to ARA in the management of symptomatic DDwR.

## Materials and methods

### Trial design

To address the study purpose, the investigators designed and performed a prospective double-blinded RCT. This trial has been registered at ClinicalTrials.gov with identification number: NCT05194488, registration date: January 2022. The authors certify that this trial has received ethical approval from the Research Ethics Committee, Faculty of Dentistry, Alexandria University, Egypt (international No.: IORG0008839, ethics committee number: 0399–2/2022; date of registration: 1/2/2022). Signed written informed consent forms were obtained before enrollment in this trial.

### Sample size calculation

Based on data from previous studies [14, 17, 34] a sample size of 27 subjects was calculated using the G*Power software program version 3.1.9.6 (Heinrich-Heine-Universität Düsseldorf, Germany) with 80% power and a 5% significance level **α** error [46].

### Inclusion and exclusion criteria

This study was conducted in Temporomandibular Dysfunction Clinic at the Prosthodontic Department, Faculty of Dentistry, Alexandria University. All subjects enrolled had painful TMJ with clicking. The subject-related inclusion criteria were: (i) TMJ noise during jaw movement or function; (ii) age range between 20 and 40 years; (ii) Angle class I maxillo-mandibular relation. Subjects were excluded if they had: (i) a history of recent oral, facial,or cervical trauma; (ii) 5 ≥ un-restored missing posterior teeth; (iii) anterior open bite; (iv) myofascial pain or systemic disease that could influence the masticatory system, bone, and joints (e.g., epilepsy, osteoarthritis, and rheumatoid arthritis); (v) received any treatment for TMD; (vi) Allergy to BTX; (vii) any neurological disorders (Viii) signs of degenerative changes in joint and/or non-reducible disc on MRI; (ix) contraindication to be exposed to MRI examination (e.g., patients with pacemakers, intracranial vascular clips, and metal particles in vital structures); and (x) pregnant or lactating females. According to the previous strict inclusion and exclusion criteria, patients were selected and examined clinically through the DC/TMDs criteria by an experienced blinded examiner. Only 27 patients diagnosed as DC/TMDs Axis I group II.a indicating DDwR and confirmed by MRI were enrolled in the study.

### Randomization

Each patient included in the current study was given a serial number that was used in the allocation. A duplicate of these numbers was written down and placed in opaque sealed envelopes with the respective names of patients. A trial independent analyst assigned the patients randomly to 3 equal groups; ARA group (I), BTX-A group (II), and LLLT group (III), using a computer-generated list of random numbers. Randomization sequence in blocks of 3 was created using randomization software (Sealed Envelope, London, UK). The allocated group was written down on a piece of paper that was enfolded in an opaque sealed envelope with the patient’s respective number. At intervention time, an assistant opened the envelopes and identified the group to which the patient was allocated.

### Interventions

Patients in group I, the control group (*n* = 9), received a hard full-arch maxillary ARA fabricated from clear acrylic resin according to Okeson’s technique [12]. All the patients were instructed to wear the ARA at night for the entire treatment period of 3 months.

**Fig. 2:**
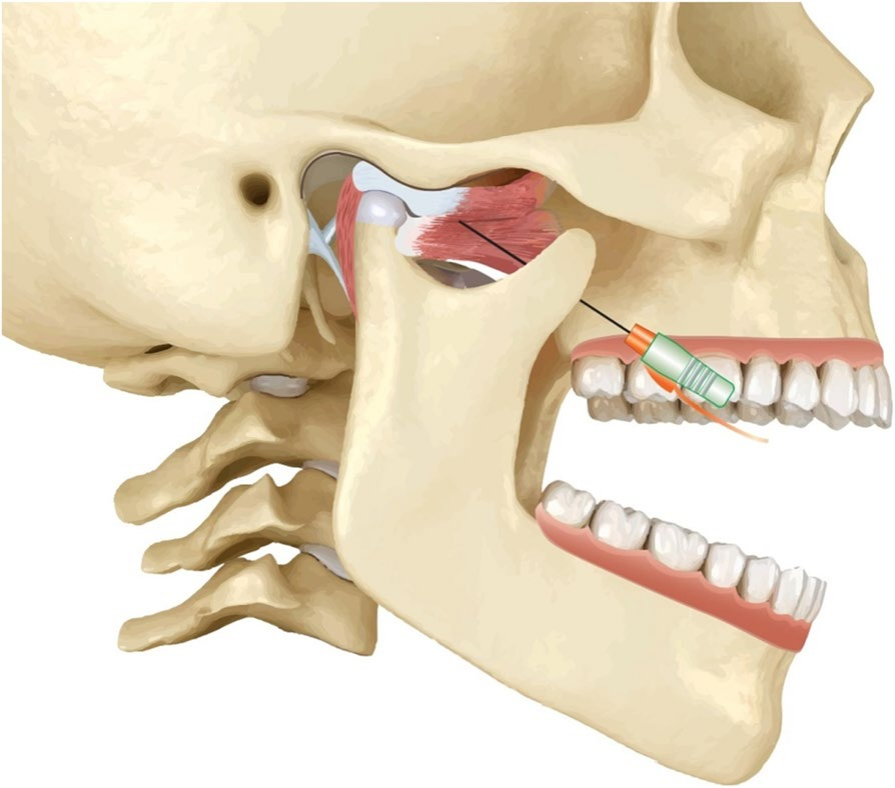
Illustrative diagram of BTX-A injection technique to the LPM

**Fig. 3:**
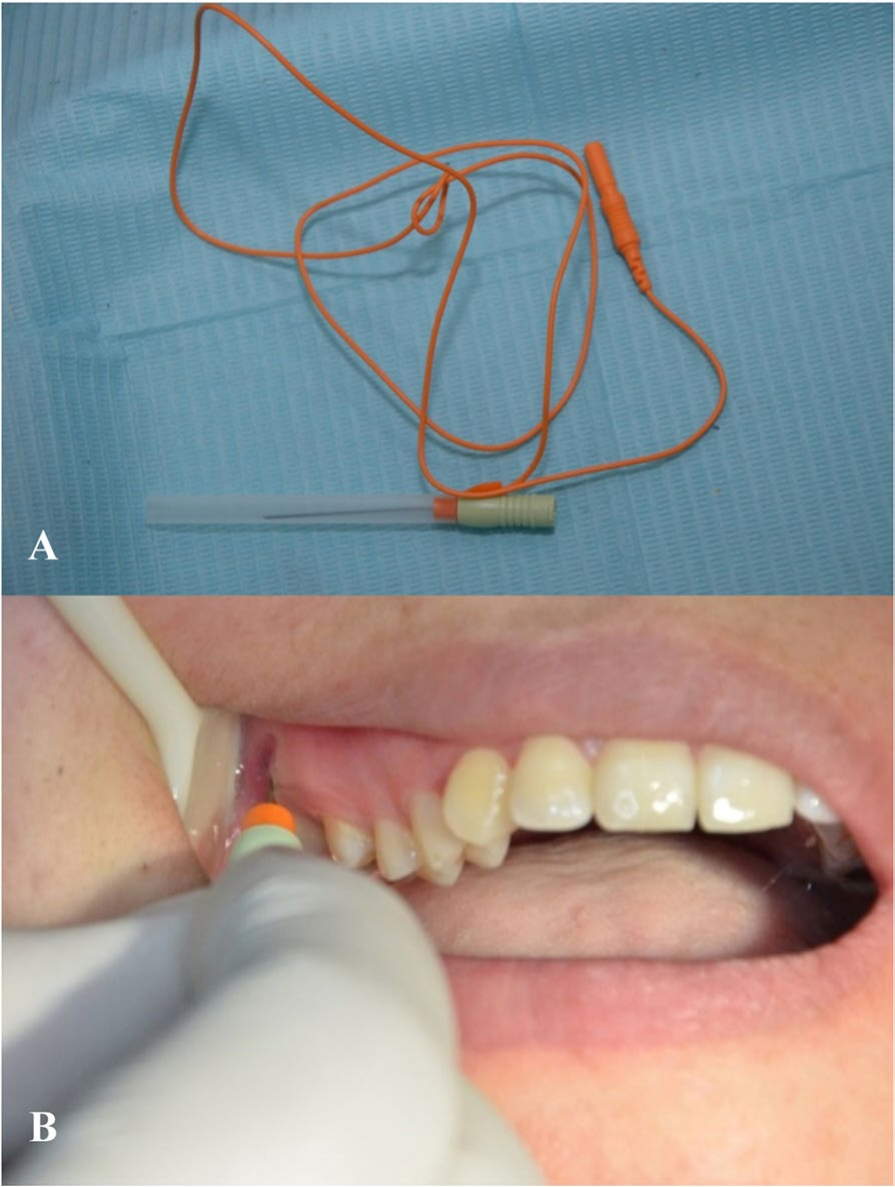
EMG Needle electrode. **A.** 27-gauge monopolar cannulated needle electrode, **B.** The EMG needle electrode in site of injection intra-orally

For group II, a single injection of 30 units of BTX-A (Botox; Allergan, CA) under electromyographic guidance (Dantec Clavis; Natus, USA) was done using an intraoral approach to the LPM without differentiation between both heads (Fig. 2). A 27-gauge monopolar cannulated needle electrode was inserted lateral to the maxillary tuberosity, just above the maxillary molars (Fig. 3A, B). Then the patient was asked to manipulate the mandible to activate the LPM. The intramuscular injection was confirmed when the electromyographic device produced a distinct loud sound [9, 36].

**Fig. 4:**
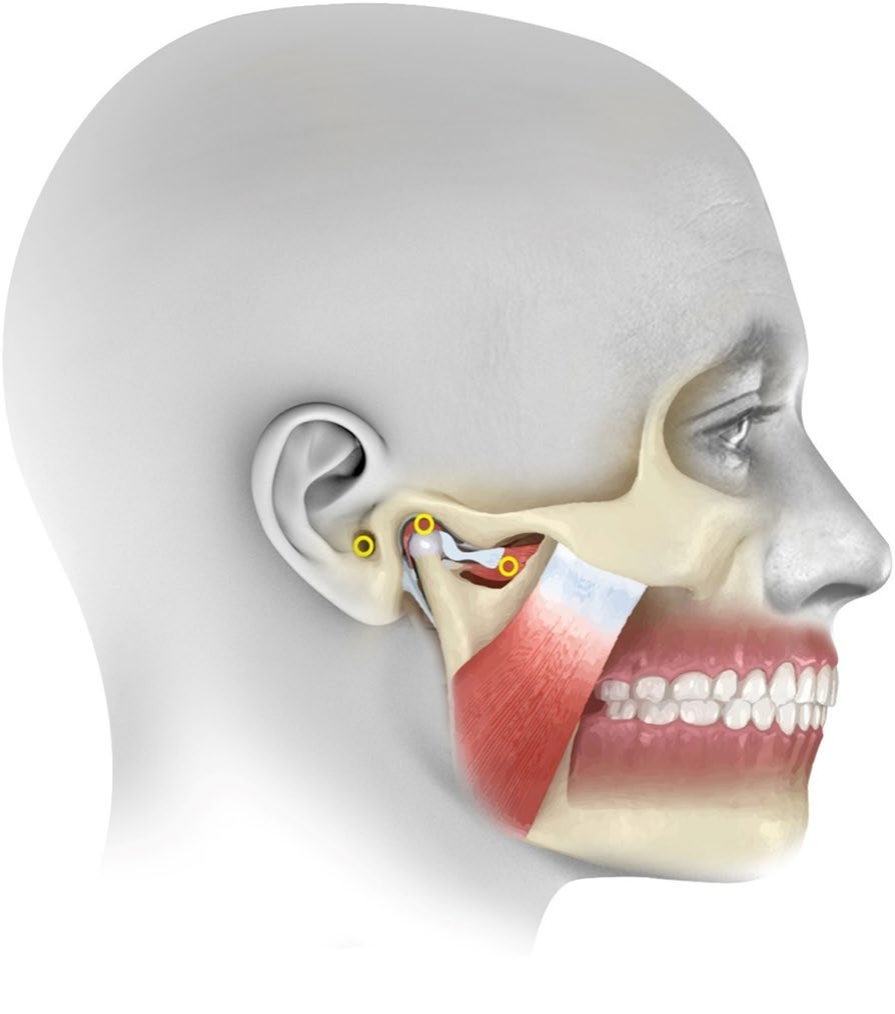
Illustrative diagram of LLLT applications points

For group III, the LLLT was applied using Endolaser (Gallium-Aluminum- Arsenide. ENDOLASER 476, Enraf Nonius, Netherlands.). This diode laser emits a continuous laser beam of 780 nm wavelength with 100mW power output, and energy density was adjusted to 1.4 J/cm2 for one and half minutes over 3 trigger points at TMJ; the posterior aspect of the joint; the area anterior to condyle 1 cm from the tragus; and at the joint area in the opened mouth position [47] (Fig. 4).

The patient and investigator wore protective goggles during the Laser application. The LLLT was applied 3 times per week for the entire treatment period.

### Assessment

Patients were evaluated by an experienced outcome assessor who was blinded to the study at the beginning of treatment and 3 months after the treatment. All patients were evaluated subjectively through VAS to clinically assess the pain intensity with ratings between 0 for pain-free and 10 for maximum pain. Time of recovery in terms of reduction in pain intensity and/or cessation of clicking was noted during the evaluation period. Furthermore, an objective assessment of the articular disc position, as well as the condylar position, was assessed by MRI. All patients underwent MRI using a 3 Tesla MRI scanner, with a head coil, in closed and open mouth positions in the oblique sagittal plane using gradient T2 and proton-density weighted spin-echo sequences. Same MRI slices were selected and compared to the baseline ones. A specific software program (PaxeraViewer, version 1.0.0.8, PaxeraMed Corp.) was used to measure the disc position by applying Kurita et al. method [48] and condylar position using Rammelsberg et al. method [49].

### Measurement of articular disc position

**Fig. 5:**
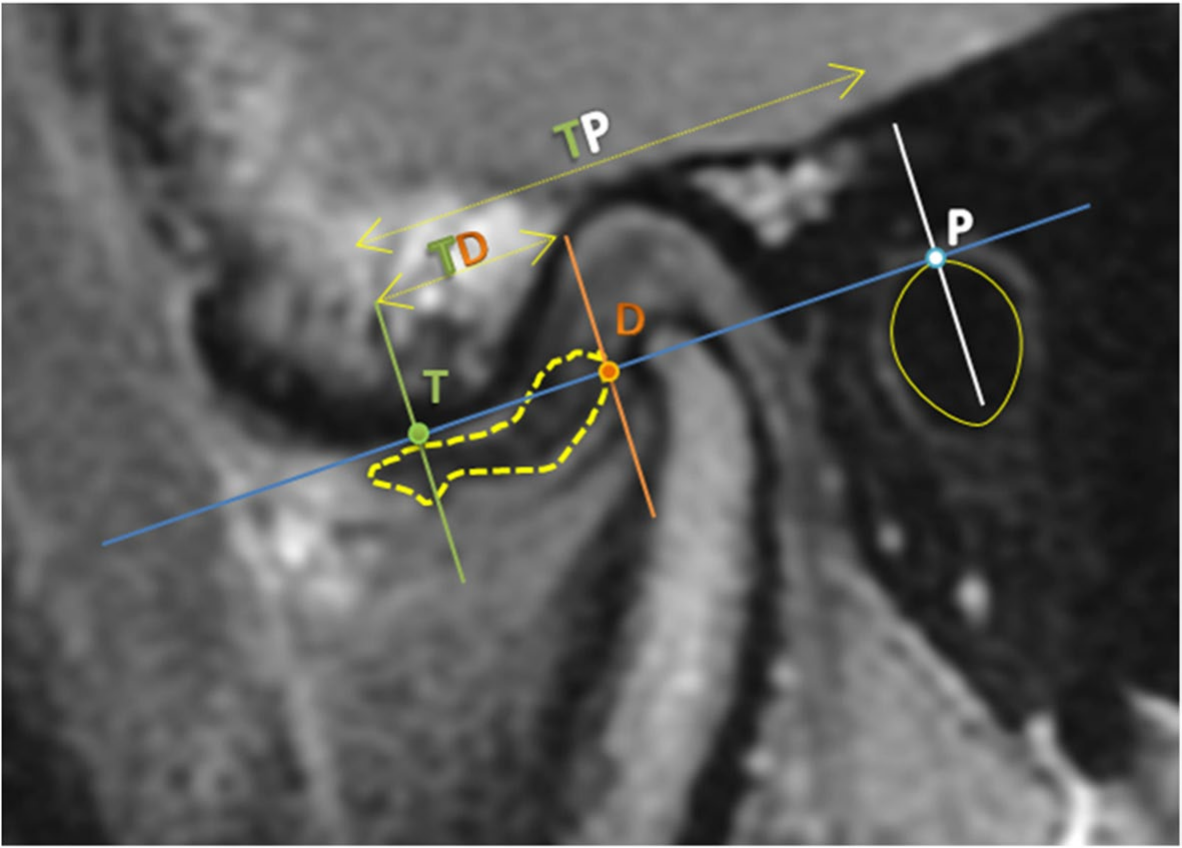
Application of Kurita et al. method on MRI for measuring the disc position

A tangent was drawn between the lowest point of the articular eminence (T) and the highest edge of the external auditory canal (P). Perpendicular to the tangent, a line was drawn from the posterior end of the disc intersecting it at point D. The TP and TD distances were measured in millimeters then the relative disc location to TP was calculated as TD/TP (Fig. 5).

### Measurement of condylar position

The center of the condyle was detected to coincide with that of an imaginary circle corresponding to the condyle outline. A reference line was drawn parallel to the Frankfort plane passing through the condyle center. The area between the lowest signal intensity of condyle and fossa within an angle of 45 anteriorly and posteriorly from the center point was calculated as JSI:

$$JSI=\frac{Post-Ant }{Post+Ant} X 100$$

**Fig. 6:**
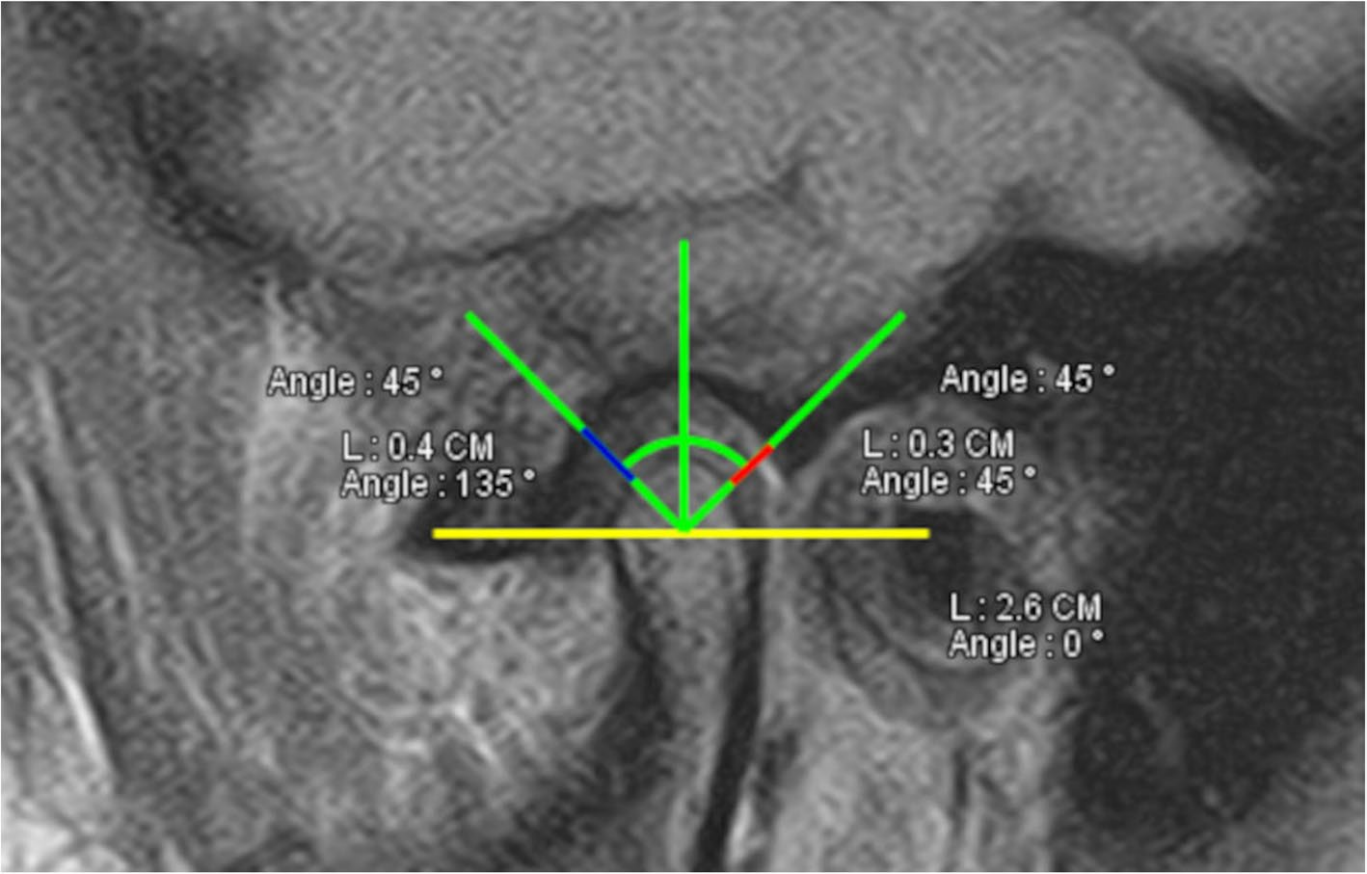
Application of Rammelsberg et al. method on MRI for measuring the condylar position

A positive value indicates anterior condylar position, while a negative value reflects the posterior condylar position within the fossa. (Fig. 6).

### Blinding

Blinding of the investigator and the patients was impossible to be done during the trial due to the nature of the intervention; however, the outcome assessor who did the follow-up assessments did not know to which group the patient belonged. Furthermore, the data analyst did not know to which group the data belonged; hence, double-blinded trial.

### Statistical analysis

Collected data were fed to the computer and analyzed using SPSS software package version 20.0. (Armonk, NY: IBM Corp) [50].

Chi-square test was used to investigate the association between the categorical variables. Alternatively, Monte Carlo correction test was used when the expected counts were less than 5. Shapiro–Wilk test was used to determine the normality of continuous data. For normally distributed quantitative variables, paired t-test was used for comparison between 2 periods, while ANOVA was used for comparing the 3 groups, then Post Hoc test (Tukey) for pairwise comparison. On the other hand, for non-normally distributed quantitative data, Wilcoxon signed ranks test was used to assess comparison between 2 periods, while Kruskal Wallis test was used to compare different groups, followed by Post Hoc test (Dunn’s for multiple comparisons test). A *P* value < 0.05 was defined as statistically significant.

## Results

### Subjects enrolled

**Fig. 7:**
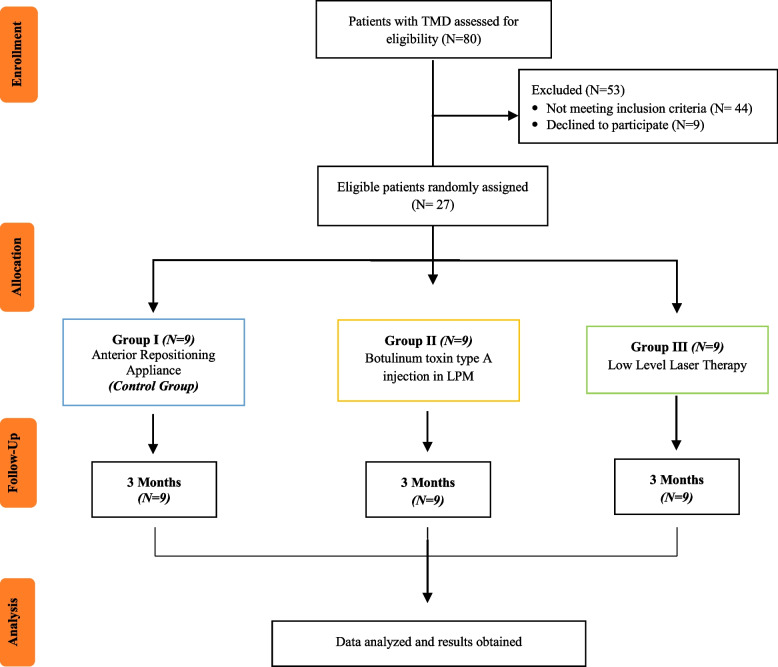
CONSORT Flow chart of the study. N, number of patients; LPM, lateral pterygoid muscle

At the beginning of this trial, the sociodemographic characteristics of patients were assessed, and no significant difference was noted (Table 1). Based on strict inclusion and exclusion criteria, 27 patients were included in this clinical trial. After a 3-month follow-up, the data were collected and applied for the statistical analysis, as shown in the CONSORT diagram (Fig. 7). All patients completed the study. Patients receiving BTX-A showed diminished contra-lateral mandibular movements after injection, with no other side effects noted.

**Table 1:** Sociodemographic characteristics of the patients and TMD symptom duration

***Characteristic***	**Group I *N* = 9**	**Group II *N* = 9**	**Group III *N* = 9**	*P*
***Age (years)***				
Mean ± SD	24.22 ± 2.9	23.22 ± 2.1	23.22 ± 2.1	0.633
***Gender (%)***				
Male	1 (11.1)	1 (11.1)	0 (0)	^MC^p = 1.000
Female	8 (88.9)	8 (88.9)	9 (100)	
***Marital status (%)***				
Married	8 (88.9)	9 (100)	8 (88.9)	^MC^p = 1.000
Single	1 (11.1)	0 (0)	1 (11.1)	
***Educational level (%)***				
High School or below	0 (0)	0 (0)	0 (0)	
College or above	9 (100)	9 (100)	9 (100)	
***Working status (%)***				
Student	2 (22.2)	2 (22.2)	2	^MC^p = 1.000
Housewife	-	1 (11.1)	-	
Working	7 (77.8)	6 (66.7)	7 (77.8)	
***TMD symptom duration (months)***				0.831
Mean ± SD	12.67 + 4.42	13.33 + 3.77	12.67 + 4.42	

Note: Group I, ARA splint therapy; Group II, BTX-A; Group III,LLLT; *SD* Standard deviation, *P*, compared between different study groups, analyzed by One way ANOVA test for age; Chi square and Monte Carlo for gender, marital and working status; Kruskal Wallis test for TMD symptom duration

#### Visual Analogue Scale of Pain (VAS)

**Fig. 8:**
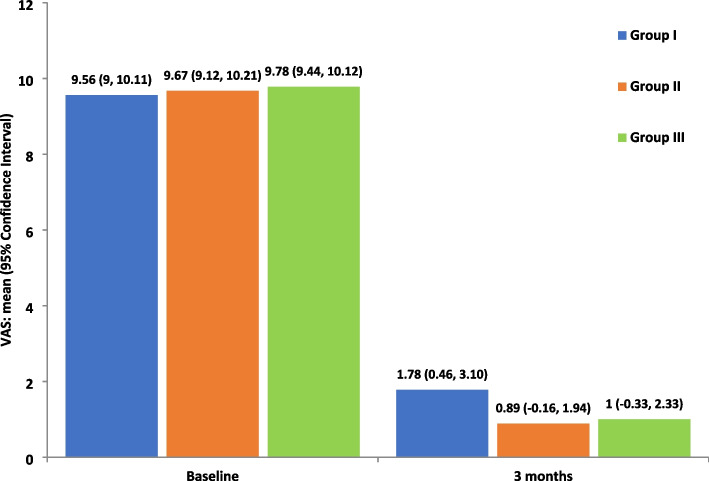
Change in pain levels over time in the study groups, measured on a 0 – 10 VAS (*P* < 0.05) (group I: ARA splint therapy; group II: BTX-A; group III: LLLT)

As pain was considered the main symptom of enrolled patients, all patients underwent subjective evaluation using VAS. There was no statistically significant difference between VAS scores in the 3 groups at the beginning of the study. After 3 months, the 3 groups showed a statistically significant reduction in pain (*P* = 0.007) with the same decrease value in both studied groups (group II and III), followed by the control group (group I). However, there was no statistically significant difference in VAS scores between the 3 groups (*P* = 0.317) (Fig. 8).

#### Measurement of articular disc position

**Fig. 9:**
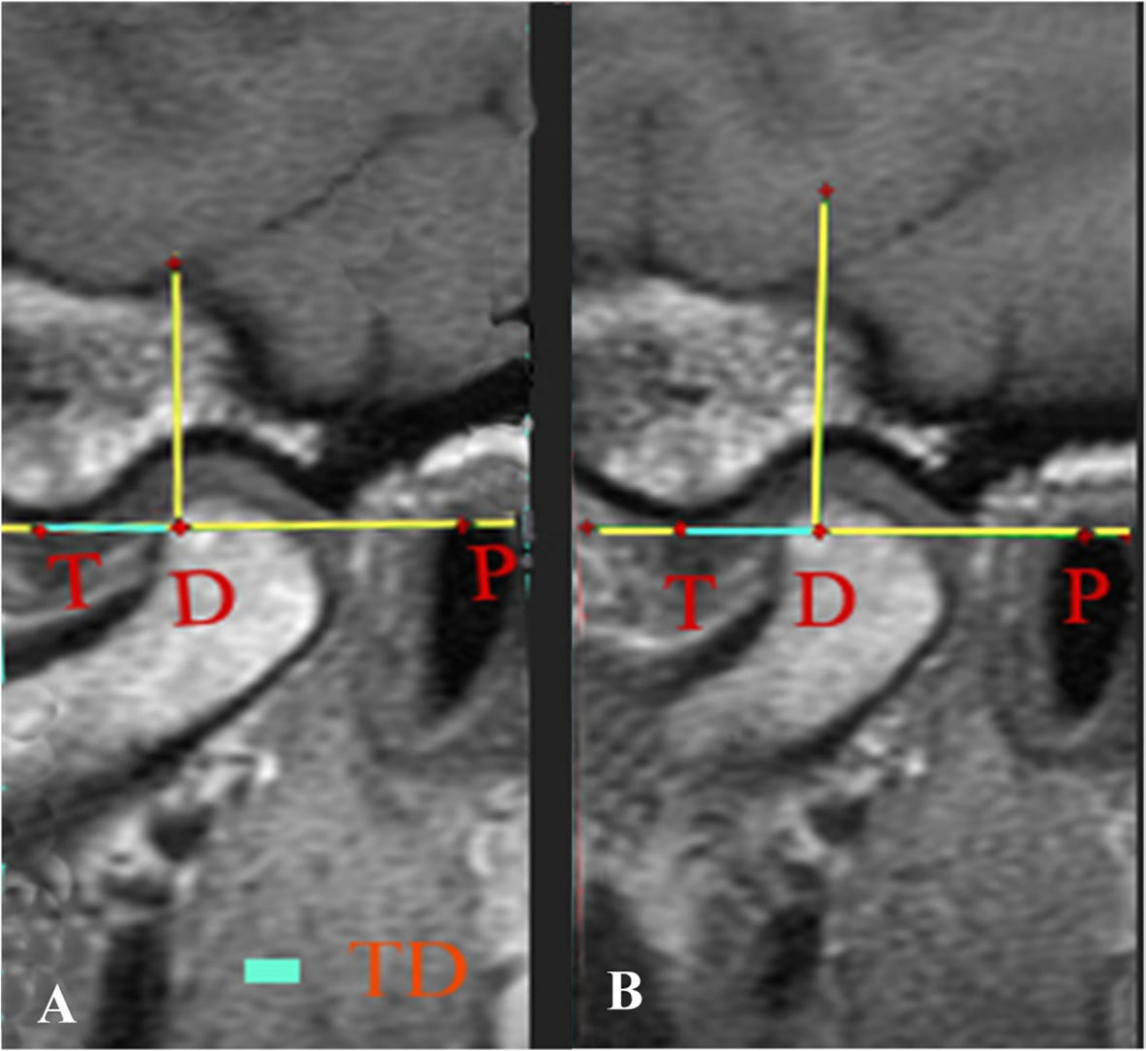
MRI showing the improvement in disc position. **A.** Before LLLT, **B.** After LLLT

**Fig. 10:**
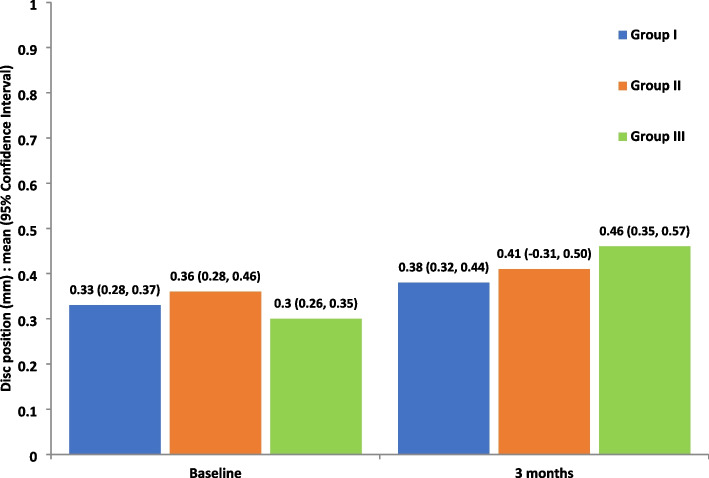
Change in disc position over time in the study groups, measured on MRI (*P* < 0.05) (group I: ARA splint therapy; group II: BTX-A; group III: LLLT)

Measured on MRI, there was an increase in the mean of TD/TP values at the evaluation time in all groups, however, group III showed the greatest mean difference (+ 0.15) (Fig. 9). Furthermore, we observed a statistically significant change in groups II and III (*P* < 0.001, = 0.002 respectively); however, there was no significant change in the mean of TD/TP values between the 3 groups (*P* = 0.059) (Fig. 10).

#### Measurement of condylar position

**Fig. 11:**
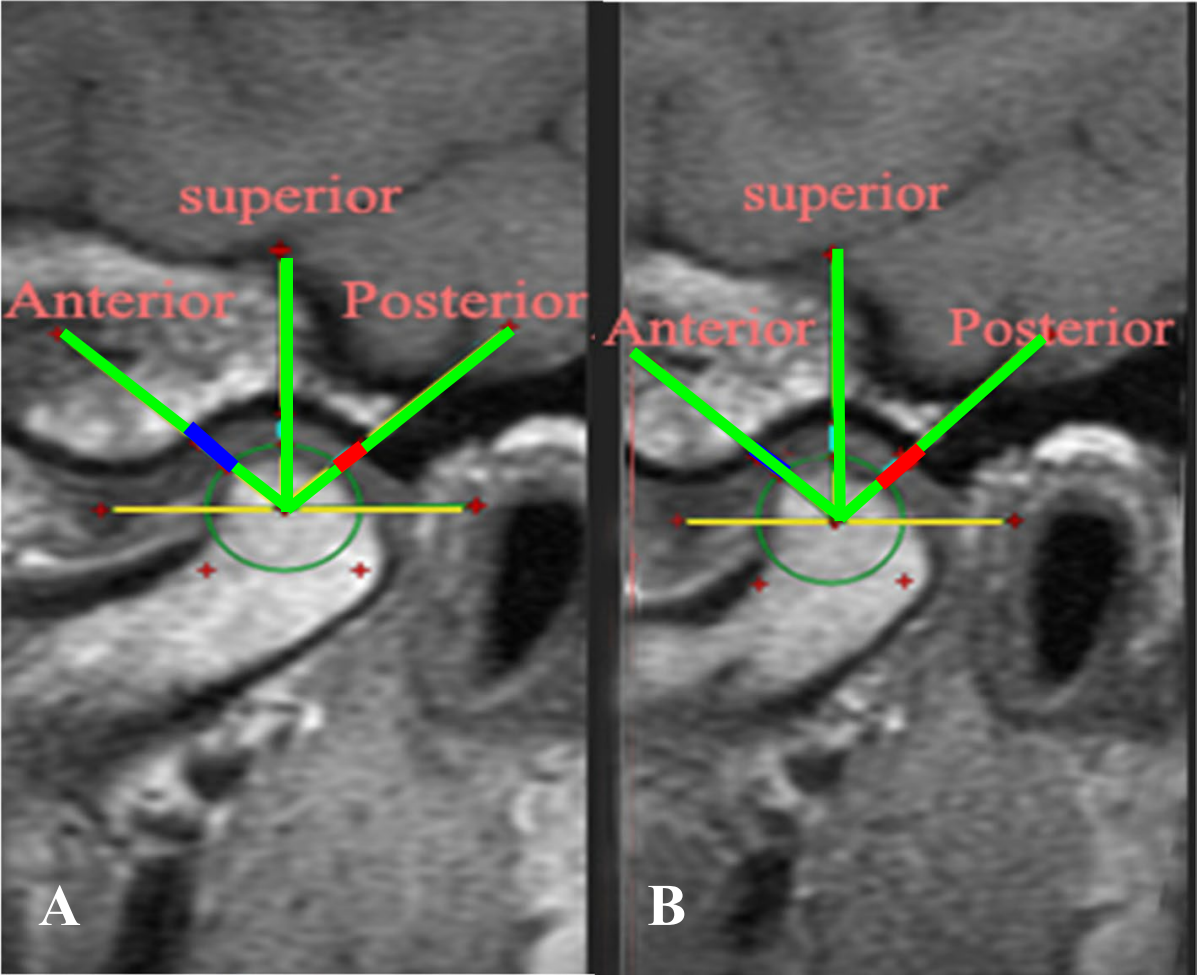
MRI showing the improvement in condylar position. **A.** Before BTX-A injection, **B.** After BTX-A injection

Although there was a change in the condylar position in the 3 groups after 3 months as measured on MRI, we found a significant difference only in the studied groups II and III (*P* = 0.011, = 0.017 respectively), while in the control group, the difference was statistically insignificant (*P* = 0.066). The change was calculated with the highest value for group II (37.0), followed by group III (25.98), and then group I (17.65) (Fig. 11). However, the change was insignificant between the 3 groups (*P* = 0.097). Furthermore, JSI was of a negative value in all groups indicating a posterior condylar position within the glenoid fossa (Table 2).

**Table 2:** Measurements of condylar position as JSI in each group, data was expressed using mean ± SD

	**Pre**	**Post**	***P***
Group I	-37.81 ± 7.50	-20.26 ± 19.95	0.066
Group II	-40.67 ± 7.91	-3.67 ± 11.0	0.011^*^
Group III	-33.52 ± 7.62	-7.54 ± 15.61	0.01^*^

Note: *JSI* Joint Space Index, *SD* Standard deviation, *Group I* ARA splint therapy, *Group II BTX-A* Group III, LLLT; *SD* Standard deviation*, p*, compared between different study groups, analyzed by Wilcoxon signed ranks test; *: Statistically significant at *P* ≤ 0.05

#### Time of recovery

**Fig. 12:**
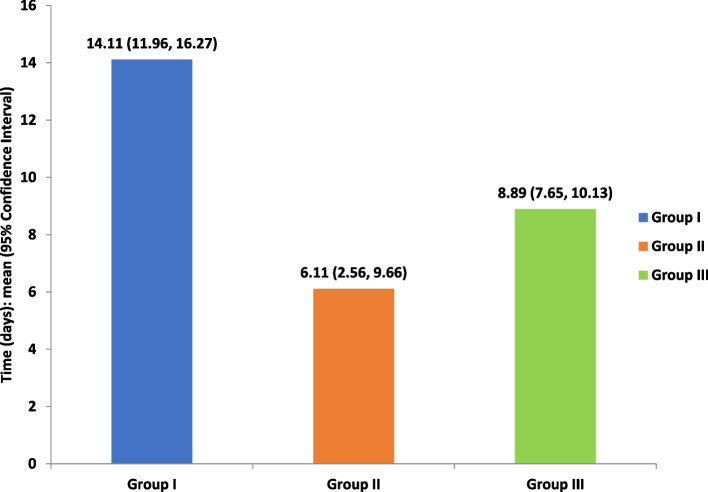
Comparison between the three groups regarding the time of recovery through the 3 months of evaluation (*P* < 0.05), (group I: ARA splint therapy; group II: BTX-A; group III: LLLT)

A statistically significant difference was observed regarding the 2 studied groups (groups II and III) in comparison to the control group (*P* < 0.001); however, the difference between the studied groups was insignificant. Furthermore, group II showed the least mean value (6.11), indicating a rapid relief of symptoms, followed by group III (8.89) in comparison to the control group, which showed the most prolonged duration required for relief of symptoms (14.11) (Fig. 12).

## Discussion

ID is the most prevalent sub-classification of TMDs that is often referred to our clinic of Prosthodontic Department. In this prospective RCT, patients with DDwR were enrolled. The highest proportion of patients was among females aged 20–40 years, as the middle-aged individuals showed a higher incidence of TMDs signs and symptoms. In addition, women have lower levels of muscle strength under stress than men [51–53]. Only patients with Angle class I maxillomandibular relation were enrolled in this study to avoid abnormal stresses which might be exerted in other classes [54].

As DDwR is a multifactorial disorder, patients with a history of recent trauma were excluded from the study. Similarly, patients with ≥ 5 un-restored missing posterior teeth or having an anterior open bite were not enrolled as these occlusal features may contribute to TMDs. Patients with a previous history of receiving any TMDs treatment were not selected to avoid biased results. In accordance with the BTX manufacturer recommendations, pregnant and lactating females, patients with allergy to BTX, or those having any neurological disorders were excluded.

Proper diagnosis is the key to successful treatment; therefore, a thorough diagnosis was done through history questionnaires and clinical examination according to DC/TMD and TMJ imaging [2, 10, 55, 56].

MRI was chosen as an imaging technique to confirm DDwR as it is considered the gold standard for imaging TMJ providing information on soft and hard structures of the joint [57]. Patients showing any degenerative changes of the TMJs on MRI were excluded. These changes might impair the measurements for evaluating disc position since the measurement method used depends on intact osseous reference points to gain valid and reliable results [58]. Patients contraindicated to be exposed to MRI were excluded as the magnetic field may dislodge the metallic objects leading to undesirable bleeding or impairment in pacemaker function [59].

Several types of oral appliances are used to treat DDwR; however, ARA is the recommended appliance for treating such patients, as documented in the literature [19–22, 60–62]. Summer and Westesson [19] stated that ARA is effective in the treatment of DDwR. Williamson and Rosenzweig [20] stated that wearing ARA could help patients prevent progression from clicking to locking “non-reducible disc” and permit retrodiscal tissues to heal. Based on these clinical studies, ARA was used as a treatment modality for the control group in the current study.

ARA can be used for both arches. However, the maxillary arch is preferred as the palatal guiding ramp can be more easily fabricated to direct the mandible into the desired protruded position. In addition, as the maxillary ARA was intended for night-time use, the palatal guiding ramp restricts the retrusion of the mandible during sleep [12].

Many methods have been suggested for the fabrication of ARA. In the current study, Okeson’s technique was used, in which the precise protruded mandibular position was located directly intraorally, thus minimizing the laboratory steps with accompanied inaccuracies and proceeding with the treatment without delay [12].

Patients were advised to wear the ARA only at night firstly; to limit the adverse effects on the occlusion as posterior open bite, resulting from myostatic contracture of LPM, and secondly, the normal function of condyle during the day promotes the development of fibrotic connective tissues in the retrodiscal tissues [12, 16, 17, 63]. Consequently, with this part-time use of ARA, no side effects were observed in any patient after 3 months.

With the evolvement of minimally invasive treatment modalities for DDwR, the need to test their effectiveness compared to ARA was important. The null hypothesis was partially accepted as there was no statistically significant reduction in pain assessed by VAS scale. Similarly, there was no statistically significant difference in both articular disc and condyle position in all groups, as shown in MRI. While in terms of time of recovery, there was a statistically significant difference.

In patients receiving ARA (group I), the disc position was improved, which goes in agreement with Kurita et al. [58]. After the treatment, 22% of the patients were pain-free; this may be due to the slight improvement in disc position detected by MRI in addition to the healed retrodiscal tissues that acted as pseudo-disc. This result was in conformance with the studies verifying the effectiveness of ARA in patients with DDwR [17–22, 64].

An interesting finding was observed in group II in which 22% of the patients showed a reduction of pain and cessation of clicking 1 day after BTX-A injection to the LPM. This contrasted with Hassan et al. study [36], in which the effect of BTX-A took 2 weeks to appear. However, our result was in agreement with the fact that the onset of BTX-A occurs within 7 days and even may appear in the first 24 h [61]. This may be attributed to the difference in muscle response to injection as BTX-A is dose-dependent and affected by the muscle mass, which was also suggested by Lorenc et al. [65] and Schwartz and Freund [66].

The change in the disc position as observed in MRI after injection of BTX-A without differentiation between the 2 heads of the LPM during injection supported Murray et al. proposal that inferior and superior heads of LPM should be considered as a system of fibers that act as one muscle, with the distribution of muscle activity being controlled by the biomechanical demands of the task [67]. Furthermore, it was in accordance with Schwartz and Freund, who stated that the location of BTX-A injection diffusely affects the whole muscle [66]. As BTX-A inhibits the release of acetylcholine in presynaptic junction it produces a reduction in muscle activity, thus the observed change in the disc-condyle relationship could be attributed to the BTX-A effect on the LPM activity after injection. Moreover, it supported considering the LPM as a possible etiology of DDwR where the inferior head of the LPM indirectly controls the disc position in relation to the maxilla, as suggested by Kiliç et al. [68].

In addition, these results went in agreement with those obtained from Bakke et al. [9] and Hassan et al. [36] studies, who reported improvement of the TMD symptoms after BTX-A injection with a slight improvement in disc position shown in MRI.

Regarding the side effects after administration of BTX-A, patients manifested diminished contra-lateral mandibular movements after injection, and no dysphagia was noted. This was in accordance with Bakke et al., who reported the same manifestation in their study [9]. However, this was in disagreement with Hassan et al. [36] who reported a single case of dysphagia that disappeared 5 weeks post-injection.

In group III, photobiomodulation was performed using a diode laser of 780 nm wavelength 3 times per week for the entire treatment period. By the end of treatment, 67% of patients were pain-free. Moreover, a significant improvement in VAS scores was found, which was in agreement with Hosgor et al. [14], Kulekcioglu et al. [69], and Venancio et al. [47].

Within 8 days, clicking disappeared in 33% of patients, which may be contributed to the therapeutic effect of LLLT. This finding was in accordance with Hansen and Thorøe [70], who observed a significant effect of LLLT on TMJ clicking. However, this finding was in disagreement with Hosgor et al. [14], who reported no change in TMJ clicking. By the end of treatment, no clicking was noted due to the change in disc position, which was interpreted on MRI in 78% of the patients. The observed change in the disc-condyle relationship may be attributed to the photobiomodualtion effect on the micro-environment of the TMJ and muscular attachment to the disc.

Regarding the therapeutic effect, a significant shortening in the time needed for recovery was observed in all groups. However, the patients who received BTX-A injection showed faster relief in symptoms within 1 week, followed by the patients receiving LLLT who showed relief within 8 days, while in the ARA group, symptoms were relieved within 2 weeks. This finding may be attributed to the direct targeting of TMJ via photobiomoduation by LLLT, while BTX-A acts as a local LPM muscle relaxant. Moreover, BTX-A and LLLT inhibit acetylcholine release, which contributes to the rapid relief of pain.

The point of strength of our study is that it was, to the authors’ knowledge, the first RCT to compare objectively and subjectively the effectiveness of BTX-A and LLLT to ARA in managing symptomatic DDwR.

The limitation of this RCT was the short follow-up period restricted to 3 months which was the safe period to use ARA without the occurrence of any adverse effects. More RCTs are needed with longer follow-up periods to allow for remodeling of TMJ tissues which could be interpreted by MRI as an improvement in the disc-condyle relationship.

## Conclusions

Based on the results of this RCT, the 3 treatment modalities were effective in the treatment of DDwR. However, owing to the pitfalls of ARA regarding its short-term use, dependence on patient’s compliance, and its potential side effects, BTX-A and LLLT could be considered effective alternatives for managing patients with symptomatic DDwR. We found that BTX-A and LLLT resulted in a significant reduction in pain with rapid relief of symptoms as well as statistically significant change in disc-condyle relationship. Future RCTs are needed on the therapeutic effects of different doses of BTX-A and various parameters of LLLT in management of symptomatic DDwR.

## Data Availability

The datasets used and analyzed during the current RCT are available from the corresponding author upon reasonable request.

## Abbreviations

DDwR: Disc displacement with reduction
RCT: Randomized controlled trial
BTX-A: Botulinum toxin type-A
LLLT: Low level laser therapy
ARA: Anterior repositioning appliance
VAS: Visual analogue scale
MRI: Magnetic resonance imaging
TMDs: Temporomandibular disorders
TMJ: Temporomandibular joint
ID: Internal derangement
LPM: Lateral pterygoid muscle
DC/TMDs: Diagnostic criteria for temporomandibular disorders
BTX: Botulinum toxin
JSI: Joint space index
